# Mental health screening and increased risk for anxiety and depression among treatment-seeking smokers

**DOI:** 10.1186/1617-9625-12-20

**Published:** 2014-11-04

**Authors:** Nilufer Emre, Kenan Topal, Nurgul Bozkurt, Eylem Topaktas

**Affiliations:** Department of Family Medicine, Pamukkale University Faculty of Medicine, C103, 20070 Kinikli, Denizli, Turkey; Denizli State Hospital, Chest Diseases Clinic, No 1, 20010 Denizli, Turkey

**Keywords:** Smoking cessation, Nicotine addiction, Mental health, Anxiety, Depression

## Abstract

**Background:**

The aim of this study was to compare the risk for mental health disorders between smokers and non-smokers and to assess the risk for depression and anxiety according to addiction severity.

**Methods:**

This cross-sectional study assesses the mental health status and relationship with the severity of nicotine addiction in a sample of smokers admitted to Pamukkale University Hospital Smoking Cessation Clinic (n = 101) from 1 June 2012 to 31 August 2012 compared to a group of non-smokers from the general population (n = 101). We conducted semi-structured face-to-face interviews to collect sociodemographic data; we assessed the participants’ mental health status with the General Health Questionnaire-12 (GHQ-12) and the Hospital Anxiety and Depression Scale (HADS), and we measured nicotine addiction severity with the Fagerström Test.

**Results:**

The risk for mental illness reported by smokers based on the GHQ-12 was significantly higher than that for non-smokers (p = 0.001). The anxiety and depression scores according to HADS were higher among smokers (16.8% and 22.8%, respectively) than non-smokers (4.0% and 5.0%, respectively) (p = 0.006 and p = 0.001, respectively). The nicotine addiction severity was higher in smokers with higher anxiety and depression scores (p = 0.008).

**Conclusions:**

We found high scores for mental illness in treatment-seeking smokers compared with non-smokers. The risk for anxiety and depression was higher among smokers. Increased nicotine addiction severity was associated with increased risk for mental illness and increased scores of anxiety and depression.

## Background

Tobacco is the single most preventable cause of death in the world today, and it is the leading cause of disease, disability, and death in both developed and developing countries. According to The World Health Organization’s Global Status Report on Noncommunicable Diseases 2010, tobacco kills nearly 6 million people per year. As the leading behavioural risk factor for noncommunicable diseases, smoking is estimated to be responsible for approximately 71% of all lung cancer deaths, 42% of chronic respiratory disease cases and nearly 10% of cardiovascular disease cases [1–3].

The best method for reducing tobacco-related morbidity and mortality is to quit smoking. Professional help and medical treatment significantly increase the cessation rates. Smoking cessation clinics are effective for smoking cessation [4–8]. In Turkey, there are more than 300 smoking cessation clinics, and in general, pulmonologists work at these clinics. Assessing the physiological, psychological, and social factors is important for success, so the health professionals who work in smoking cessation clinics should be well-trained. Additionally, it is important for the health professionals working in smoking cessation clinics to have a multidisciplinary perspective.

Smokers have a higher mental illness risk than non-smokers; therefore, it is important to screen smokers for mental illness. Breslau et al. [4] demonstrated that major depression and anxiety disorder rates are higher in people with nicotine addiction, and the rates increased with the severity of nicotine addiction. They also showed that this relationship may be causal in either direction; that is, nicotine addiction may lead to major depression, and major depression can affect nicotine use [9, 10]. Black et al. [11] examined the prevalence of lifetime psychiatric disorders in cigarette smokers and non-smokers in the general population and found that smokers more often had a history of major depression, alcohol and substance addiction, agoraphobia and other personality disorders. Nicotine addiction was 4 to 6 times more frequent in people with depression. Lifetime tobacco product use was higher in people with depression than in those without. Also, there is a reported correlation between higher depression scores and increased smoking rates [11].

The aim of this study was to assess the psychological health, anxiety and depression risk in smokers who desired to quit compared to non-smokers and to look for a relationship between mental health status and nicotine addiction severity.

## Materials and methods

### Sampling

This cross-sectional study included 101 smokers who were admitted to the Pamukkale University Hospital Smoking Cessation Clinic from 1 June 2012 to 31 August 2012 and 101 age- and gender-matched non-smokers from the general population. Participants were 18 to 65 years of age. Patients with a previous diagnosis of any mental illness were excluded from the study. The researchers prepared a questionnaire to collect sociodemographic data, including income level, educational status, smoking habits, alcohol consumption, and spouse’s and children’s smoking habits. The questionnaires were completed during face-to-face interviews.

All of the participants gave written informed consent. The study was conducted according to the Declaration of Helsinki Principles and was approved by the Pamukkale University Faculty of Medicine Ethics Committee.

### Scales

The Fagerström Test for Nicotine Dependence (FTND) was used to measure the degree of nicotine dependence. Fagerström and Schneider developed this scale in 1989 to determine the degree of nicotine addiction, and this scale is often used to measure physical dependence on nicotine. The FTND is a well-validated psychometric assessment tool that consists of six questions. The total possible score ranges from 0 (no dependence) to 10 (very high dependence) and can be categorised as ‘low dependence’ (score 0–3), ‘medium dependence’ (score 4–6), or ‘high dependence’ (score 7–10) [12, 13].

The General Health Questionnaire-12 (GHQ-12) was used to assess mental health in both groups. The GHQ-12 was developed to determine the mental condition in the community and primary health care facilities. It is one of the most commonly used scales and consists of 12 items, each assessing the severity of a mental problem over the past few weeks using a 4-point Likert-type scale. Higher scores indicate worse health. Participants with an average total GHQ 12 score above the cut-off threshold (11/12) were considered potential cases of psychological disorders [14, 15]. Validity and reliability studies of the GHQ-12 in Turkey have been conducted by Kilic et al. [16, 17].

**Figure 1:**
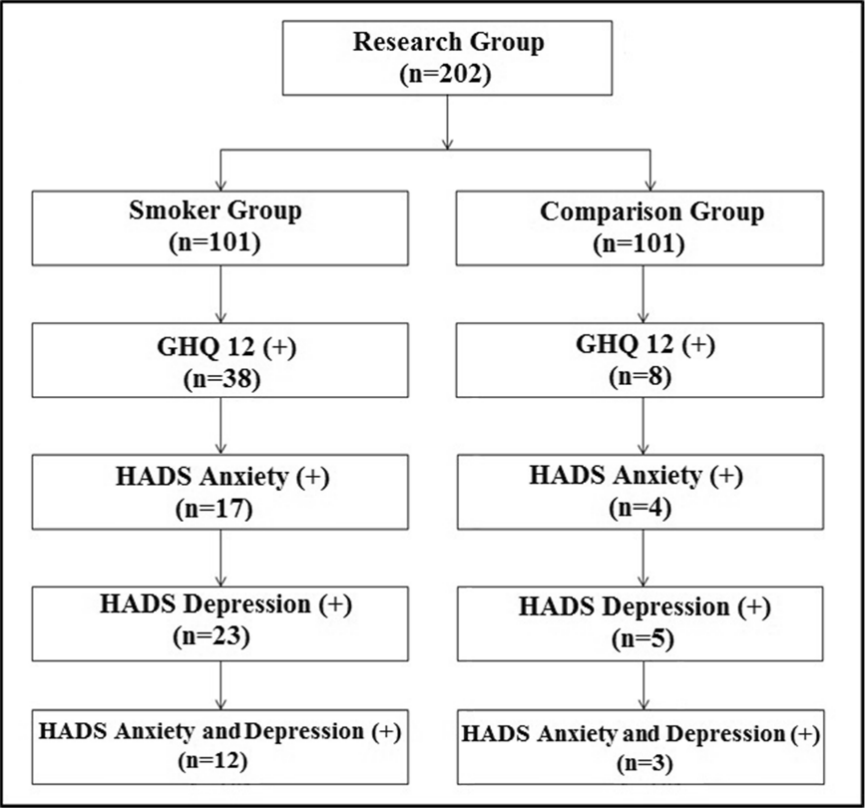
**Research flow chart.**

The Hospital Anxiety and Depression Scale (HADS) was administered to participants who were considered to be at risk for mental problems based on the GHQ-12, and it was used to assess the risk for anxiety and depression. Responses are based on the relative frequency of symptoms over the past week according to a four-point Likert scale, ranging from 0 (not at all) to 3 (very often indeed), and they are summed to provide separate scores for anxiety and depression symptomology; each anxiety or depression scale has a score ranging from 0–21. Higher scores indicate a greater likelihood of depression or anxiety, wherein a cut-off point of (10/11) is considered for Anxiety and (7/8) for the Depression subscale [18, 19]. All scales were self-administered. The flow chart of the study activities is provided in Figure 1.

### Statistical analysis

We used two independent sample t-tests for qualitative variables and Chi-Square tests or the Fisher’s exact test for categorical variables. We compared the two groups’ sociodemographic variables, including age, gender, marital status, mean income, educational status, spouse’s smoking behaviour, children’s smoking behaviour, alcohol consumption and the risk for mental health disorders based on GHQ-12 and the risk for anxiety and depression based on HADS. We analysed the relationship between gender and the level of nicotine dependency (FTND score) as well as the relationship between the risk for mental health disorders based on GHQ-12 and risk for anxiety and depression based on HADS. Logistic regression analysis (LRA) was used to estimate the simultaneous effects of age, gender, marital status, mean income, educational status, alcohol consumption and positive risk for anxiety and depression based on HADS. A two-tailed p value less than 0.05 was considered significant.

## Results

The sociodemographic characteristics of the participants are presented in Table 1. The mean age was 39.4 ± 12.7 years (range = 18-65 years), and 132 participants were male (65.3%). There were no significant differences between the smoker and comparison groups in terms of male gender (68.3% of smokers and 62.4% of the comparison group, p = 0.375) and mean age (38.9 ± 12.0 years in the smoker group and 39.9 ± 13.3 years in the comparison group, p = 0.539). There was a significant difference between the groups for marital status; 62.4% of smokers were married compared with 80.1% in the comparison group (p = 0.002). The percentage of smokers at the mean income level (31.9%) was significantly lower than that in the comparison group (49.5%) (p = 0.04). There was no significant difference between the two groups in terms of educational status (p = 0.797) (Table 1).

**Table 1:** **Sociodemographic characteristics and the risk for mental illness based on GHQ-12 and HADS**

		Smoker group (n = 101)	Comparison group (n = 101)	*X* ^*2*^	p
**Age (Year) (mean ± SD)**		38.9 ± 12.0	39.9 ± 13.3	0.6*	0.539
**Gender (Male)**		69 (68.3%)	63 (62.4%)	0.8	0.375
**Marital status (Married)**		63 (62.4%)	81 (80.1%)	14.3	0.002^†^
**Mean income**	Base wage rate or lower	46 (50.5%)	37 (40.7%)	6.5	0.040^†^
	Two or three times the base wage rate	29 (31.9%)	45 (49.5%)		
	Higher than three times the base wage rate	16 (17.6%)	9 (9.9%)		
**Educational status**	Primary school and under	49 (48.5%)	46 (45.5%)	0.5	0.797
	High school	49 (48.5%)	53 (52.5%)		
	University or higher	3 (3.0%)	2 (2.0%)		
**Spouse’s smoking**		21 (33.3%)	12 (14.8%)	6.9	0.009^††^
**Children smoking**		13 (21.3%)	6 (7.9%)	3.8	0.052
**Alcohol consumption**		36 (35.6%)	6 (5.9%)	25.3	0.001^††^
**The risk for mental illness based on GHQ-12**		38 (37.6%)	8 (7.9%)	23.7	0.001^††^
**The risk for anxiety based on HADS**		17 (16.8%)	4 (4.0%)	7.7	0.006^†^
**The risk for depression based on HADS**		23 (22.8%)	5 (5.0%)	12.0	0.001^††^
**The risk for anxiety and depression based on HADS**		12 (11.9%)	3 (3.0%)	4.6	0.032^†^

^†^p < 0.05, ^††^p < 0.01, *t-test for equality of means used for only this line, a likelihood ratio chi-square test was used for the following lines. (GHQ: General Health Questionnaire, HADS: Hospital Anxiety and Depression Scale).

The smoking status of the participants’ spouses and children was also investigated. The smoking rates were higher for the spouses and children of smokers (33.3% and 21.3%, respectively) than for the spouses and children of the comparison group (14.8% and 7.9%, respectively). The spouses’ smoking behaviour was significantly higher in smokers compared to the comparison group (p = 0.009), whereas children’s smoking behaviour was not significantly different between the groups (p = 0.052). There was a significant relationship between smoking and alcohol use. The smokers’ alcohol consumption was six times greater than that of non-smokers (p = 0.001).

The GHQ-12 scale results were significantly related to smoking behaviour.

Smokers reported a high score, above the threshold for mental illness, 4.5 times more frequently than non-smokers; 38 (37.6%) smokers reported a high score that was above the threshold for mental illness compared with only eight people (7.9%) in the comparison group (p = 0.001). Participants who reported a high score above threshold for mental illness according to the GHQ-12 completed HADS. The risk for anxiety in smoker group (n = 17, 16.8%) was higher than that of the comparison group (n = 4, 4.0%), (p = 0.006). In the smoker group, 23 people (22.8%) were at risk for depression, whereas five people (5.0%) in the comparison group were at risk for depression (p = 0.001). The risk for depression and anxiety was 3% in the comparison group and 11.9% in the smoker group (p = 0.032) (Table 1).

The mean age for starting to smoke was 18.5 ± 5.9 years (range = 7-36 years), and the mean duration of smoking was 19.7 ± 12.7 years (range = 2-48 years). According to FTND results, most smokers (n = 46, 45.5%) had a low level of dependence (p = 0.001). The degrees of addiction between genders were similar; 44.9% of men and 46.9% of women had low dependence, 26.1% of men and 31.2% of women had moderate dependence, and 29.0% of men and 21.9% of women had high dependence (p = 0.725). The GHQ-12 scores were above the cut-off threshold in 38 (37.6%) of the smokers. The rates of low, moderate, and high dependence for GHQ-12 positive smokers were 26.3%, 34.2%, and 39.5%, respectively. Among GHQ-12 negative smokers, 57.1% had low dependence, 23.8% had moderate dependence, and 19.0% had high dependence. GHQ-12 positivity and the degree of nicotine dependence were significantly related. GHQ-12 positive smokers had higher mean FTND scores (5.03 ± 2.5) than the GHQ-12 negative smokers (3.57 ± 2.9) (p = 0.011). Of the 38 smokers who reported a positive mental illness, 17 people (44.7%) were above the HADS cut-off point for anxiety (10/11) and 23 people (60.5%) were above the cut-off point for depression (7/8) in the smoker group. We found that as the level of nicotine dependence increased, the anxiety and depression scores also increased (p = 0.022 and p = 0.037, respectively) (Table 2).

**Table 2:** **Comparison of the gender, risk for mental illness based on GHQ-12 and HADS among smokers according to the Fagerström test score levels**

		Fagerström test scores (n = 101)			*X* ^2*^	P
		0-3 point	4-6 point	7-10 point		
		Low dependence	Medium dependence	High dependence		
		n = 46	n = 28	n = 27		
**Gender**	Male (n = 69)	31 (44.9%)	18 (26.1%)	20 (29.0%)	0.6	0.725
	Female (n = 32)	15 (46.9%)	10 (31.2%)	7 (21.9%)		
**GHQ-12**	Positive (n = 38)	10 (26.3%)	13 (34.2%)	15 (39.5%)	9.6	0.008^††^
	Negative (n = 63)	36 (57.1%)	15 (23.8%)	12 (19.0%)		
**HADS anxiety**	Positive (n = 17)	4 (23.5%)	5 (29.4%)	8 (47.1%)	5.3	0.022^†^
	Negative (n = 84)	42 (50.0%)	23 (27.4%)	19 (22.6%)		
**HADS depression**	Positive (n = 23)	6 (26.1%)	8 (34.8%)	9 (39.1%)	4.4	0.037^†^
	Negative (n = 78)	40 (51.3%)	20 (25.6%)	18 (23.1%)		

^†^p < 0.05, ^††^p < 0.01, *A linear by linear association chi-square test was used.

Table 3 presents the results of the multiple regression analysis that estimates the simultaneous effects of age, gender, marital status, mean income, educational status, alcohol consumption and the risk for anxiety and depression based on HADS. These analyses suggested that there were no significant effects of age, gender and educational status on smoking. Smokers are more likely to be single, widowed or divorced than married (p = 0.024), and the mean income level seems to have a limited effect on smoking (p = 0.034). Our results indicated that alcohol consumption (p = 0.000) and the risk for depression based on HADS (p = 0.019) were the most effective factors on the smoking status of the participants. There was no statistically significant relationship between the risk for anxiety based on HADS (p = 0.107).

**Table 3:** **Multiple logistic regression results estimating the factors that affect the smoking status of smokers admitted to the smoking cessation clinic from 1 June 2012 to 31 August 2012**

		Exp(B)	95.0% C.I. for Exp B		p
			Lower	Upper	
**Age (Year)**		1.004	0.97	1.04	0.799
**Gender (Male)**		0.713	0.32	1.55	0.395
**Marital status (Married)**		3.432	1.18	9.98	0.024^†^
**Mean income**	Higher than three times the base wage rate	-			0.034^†^
	Base wage rate or lower	0.339	0.08	1.29	0.115
	Two or three times the base wage rate	0.223	0.07	0.70	0.011^†^
**Educational status**	University or higher	-			0.615
	Primary school and under	1.701	0.17	16.2	0.644
	High school	1.046	0.12	8.80	0.967
Alcohol consumption (+)*		10.972	3.61	33.2	0.000^††^
**Positive risk for anxiety based on HADS**		3.414	0.76	15.2	0.107
**Positive risk for depression based on HADS**		4.728	1.28	17.3	0.019^†^

^†^p < 0.05, ^††^p < 0.01, *Alcohol use more than 2 drinks/day for males and 1 drink/day for females (1 drink = ½ oz or 15 ml ethanol).

## Discussion

We screened treatment-seeking smokers for their mental health status and used validated scales to assess whether they have a risk of developing anxiety and depression. The risk for mental illness was significantly higher in smokers than in the non-smoking comparison group participants. The risk for anxiety and depression was higher among smokers, and the mental health scores increased with the nicotine addiction severity. The following factors were independently related to smoking: being unmarried, having a high personal income, alcohol use and a high risk for depression based on HADS according to multivariate analysis.

Alcohol use and smoking are strongly associated with each other. Smokers may have a 2 to 3 times higher risk of alcohol dependence than non-smokers [20–22]. Grant et al. [23] showed that nicotine addiction was the most prevalent among individuals with a current alcohol or drug use disorder, and there was a strong association between nicotine addiction and specific mood, anxiety and personality disorders in the total population and by gender. We found that the alcohol consumption rate in the smoker group was six times higher than the comparison group.

The mental illness risk among smokers is higher than that of non-smokers. Approximately 30% of smokers are estimated to have mental illness, and depression and anxiety symptoms are observed more often among smokers than non-smokers [24–28]. Degenhard et al. [29] found that current tobacco use is associated with a range of other substance use and mental health problems. They noted that those problems were likely to reduce the success of attempts to quit smoking; therefore, the presence of other substance use and mental health problems needs to be considered with smoking-cessation treatment [29]. The rate of high scores above the cut-off threshold for mental illness according the GHQ-12 results was high (37.6%) in the smoker group, and the risk for anxiety and depression was significantly higher for smokers compared to non-smokers.

Cosci et al. [30] assessed whether subjects applying to smoking cessation clinics display a higher level of affective symptoms than smokers recruited from the general population. Their cases had significantly higher scores than controls when the rating scales assessed anxious and depressive symptoms. The cases scored higher on the anxiety subscale of the HADS (p < 0.005) than controls. Additionally, the cases had a higher mean score for the HADS depression subscale than controls, but this result was not significantly different (p = 0.1076) [30]. We found that smokers scored higher on the anxiety (p = 0.006) and depression subscales (p = 0.001) of the HADS than the comparison group in our study. Although there was a significant relationship between smoking and the risk for depression, based on HADS, according to the multivariate analysis, there was no significant relationship with the risk for anxiety.

Jamal et al. [31] compared the severity of nicotine addiction and the course of the symptoms of depression and anxiety between smokers and non-smokers. They found that heavy smoking and nicotine addiction were associated with depression and anxiety and that the depressive symptom severity was higher among heavier smokers [30]. We also found that smokers had an increased risk of anxiety and depression, which was associated with an increased severity of nicotine addiction.

Raising public awareness, reducing young people’s access to tobacco and implementing tobacco laws and smoking policies are very important for preventing people from the harmful effects of smoking. Smoking cessation is the most important, cost-effective treatment that clinicians can offer for patients who smoke [32]. However, smoking cessation can take many years, and it is a challenging process that often requires more than one quitting attempt [9].

### Limitations

This study includes some limitations with respect to the generalizability because the study does not include a large sample size. Another limitation is the comparison group. We have some concerns about whether they match the same hypothetical population as the smokers even though they are age- and gender-matched and have a similar education level.

## Conclusions

In recent years, many smoking cessation clinics have been established across Turkey. Health professionals who work in these clinics should be well-trained, and services should be provided from a multidisciplinary perspective. Our research results and those from many other studies on similar topics have shown that smokers have high scores for probable mental illness. Therefore, it is important to screen all smokers who want to quit smoking for mental health disorders using valid scales, and referring smokers who are at a high risk of mental illness to mental health professionals should always be considered.

## References

[1] World Health Organization (2008). WHO Report on the Global Tobacco Epidemic, 2008, The MPOWER Package.

[2] World Health Organization (2011). Global Status Report on Noncommunicable Diseases 2010, Description of the global burden of NCDs, their risk factors and determinants.

[3] Bartecchi CE, MacKenzie TD, Schrier RW (1994). The human costs of tobacco use. N Engl J Med.

[4] Breslau N, Kilbey MM, Andreski P (1993). Nicotine dependence and major depression. New evidence from a prospective investigation. Arch Gen Psychiatry.

[5] American Psychiatric Association (2000). Diagnostic and Statistical Manual of Mental Disorders.

[6] Uneri O, Tural U, Memik CN (2006). Smoking and schizophrenia: where is the biological connection?. Turk Psikyiatri Derg.

[7] Karlıkaya C, Öztuna F, Solak ZA, Özkan M, Örsel O (2006). Tobacco control. Turk Toraks Derg.

[8] Lam TH, Stewart SM, Ho SY, Lai MK, Mak KH, Chau KV, Rao U, Salili F (2005). Depressive symptoms and smoking among Hong Kong Chinese adolescents. Addiction.

[9] McManus S, Howard Meltzer H, Campion J (2010). Cigarette Smoking and Mental Health in England, Data from the Adult Psychiatric Morbidity Survey 2007.

[10] Breslau N, Kilbey M, Andreski P (1991). Nicotine dependence, major depression, and anxiety in young adults. Arch Gen Psychiatry.

[11] Black DW, Zimmerman M, Coryell WH (1999). Cigarette smoking and psychiatric disorder in a community sample. Ann Clin Psychiatry.

[12] Fagerström KO, Kunze M, Schoberberger R, Breslau N, Hughes JR, Hurt RD, Puska P, Ramström L, Zatoński W (1996). Nicotine dependence versus smoking prevalence: comparisons among countries and categories of smokers. Tob Control.

[13] Uysal MA, Kadakal F, Karşıdağ Ç, Bayram NG, Uysal Ö, Yılmaz V (2004). Fagerstrom test for nicotine dependance: reliability in a Turkish sample and factor analysis. Tuberk Toraks.

[14] Goldberg DP (1972). The Detection of Psychiatric Illness by Questionnaire.

[15] Özdemir H, Rezaki M (2007). General Health Questionnaire-12 for the detection of depression. Turk Psikyiatri Derg.

[16] Kılıç C (1996). The reliability and validity study of General Health Questionnaire. Turk Psikyiatri Derg.

[17] Kılıç C, Rezaki M, Rezaki B, Kaplan I, Ozgen G, Sağduyu A, Oztürk MO (1997). General Health Questionnaire (GHQ-12 & GHQ-28): psychometric properties and factor structure of the scales in a Turkish primary care sample. Soc Psychiatry Psychiatr Epidemiol.

[18] Zigmond AS, Snaith PR (1983). The hospital anxiety and depression scale. Acta Psychiatr Scand.

[19] Aydemir Ö, Güvenir T, Küey L, Kültür S (1977). Validity and reliability of Turkish version of Hospital Anxiety and Depression Scale. Turk Psikyiatri Derg.

[20] Drobes DJ (2002). Concurrent alcohol and tobacco dependence: mechanisms and treatment. Alcohol Res Health.

[21] Breslau N (1995). Psychiatric comorbidity of smoking and nicotine dependence. Behav Genet.

[22] Room R (2004). Smoking and drinking as complementary behaviours. Biomed Pharmacother.

[23] Grant BF, Hasin DS, Chou SP, Stinson FS, Dawson DA (2004). Nicotine dependence and psychiatric disorders in the United States: results from the national epidemiologic survey on alcohol and related conditions. Arch Gen Psychiatry.

[24] Anthony JC, Warner L, Kessler R (1994). Comparative epidemiology of dependence on tobacco, alcohol, controlled substances and inhalants. Basic findings from the National Comorbidity Survey. Exp Clin Psychopharmacol.

[25] Fiore MC, Bailey WC, Cohen SJ, Dorfman SF, Goldstein MG, Gritz ER, Heyman RB, Jaén CR, Kottke TE, Lando HA, Mecklenburg RE, Mullen PD, Nett LM, Robinson L, Stitzer ML, Tommasello AC, Villejo L, Wewers ME (2000). Treating Tobacco Use and Dependence. Quick Reference Guide for Clinicians.

[26] Horn K, Dino G, Kalsekar I, Massey CJ, Tennant KM, McGloin T (2004). Exploring the relationship between mental health and smoking cessation: a study of rural teens. Prev Sci.

[27] Berlin I, Chen H, Covey LS (2010). Depressive mood, suicide ideation and anxiety in smokers who do and smokers who do not manage to stop smoking after a target quit day. Addiction.

[28] Moylan S, Jacka FN, Pasco JA, Berk M (2012). Cigarette smoking, nicotine dependence and anxiety disorders: a systematic review of population-based, epidemiological studies. BMC Med.

[29] Degenhardt L, Hall W (2001). The relationship between tobacco use, substance-use disorders and mental health: results from the National Survey of Mental Health and Well-being. Nicotine Tob Res.

[30] Cosci F, Schruers KR, Pistelli F, Griez EJ (2009). Negative affectivity in smokers applying to smoking cessation clinics: a case–control study. Depress Anxiety.

[31] Jamal M, Van der Does AJW, Cuijpers P, Penninx BWJH (2012). Association of smoking and nicotine dependence with severity and course of symptoms in patients with depressive or anxiety disorder. Drug Alcohol Depend.

[32] Anczak JD, Nogler RA (2003). Tobacco cessation in primary care: maximizing intervention strategies. Clin Med Res.

